# Bioinformatics analysis of prognostic value and prospective pathway signal of miR-30a in ovarian cancer

**DOI:** 10.1186/s13048-020-00722-8

**Published:** 2020-10-01

**Authors:** Weijia Lu, Yunyu Wu, Can Xiong Lu, Ting Zhu, Zhong Lu Ren, Zhiwu Yu

**Affiliations:** 1 Guangzhou University of Chinese Medicine, No.232, Waihuandong Road, University Town, Panyu District, Guangzhou, 510006 Guangdong China; 2 Department of Gynaecological Oncology, Affiliated Cancer Hospital and Institute of Guangzhou Medical University, Guangzhou, 510095 Guangdong Province China; 3 Laboratory Department, Foshan Sanshui hospital of Traditional Chinese Medicine, Foshan, 528100 Guangdong China; 4 Division of Laboratory Science, Affiliated Cancer Hospital and Institute of Guangzhou Medical University, No.78, Hengzhigang Road, Yuexiu District, Guangzhou, 510095 Guangdong China; 5 College of Medical Information Engineering Guangdong Pharmaceutical University, Guangzhou, 510006 Guangdong China

**Keywords:** Differentially expressed genes, Functional factors;miR-30a, Ovarian Cancer

## Abstract

**Objective:**

**M**icroRNAs (MiRNAs) is thought to play a critical role in the initiation and progress of ovarian cancer (OC). Although miRNAs has been widely recognized in ovarian cancer, the role of hsa-miR-30a-5p (miR-30a) in OC has not been fully elucidated.

**Methods:**

Three mRNA datasets of normal ovarian tissue and OC, GSE18520,GSE14407 and GSE36668, were downloaded from Gene Expression Omnibus (GEO) to find the differentially expressed gene (DEG). Then the target genes of hsa-miR-30a-5p were predicted by miRWALK3.0 and TargetScan. Then, the gene overlap between DEG and the predicted target genes of miR-30a in OC was analyzed by Gene Ontology (GO) enrichment analysis. Protein-protein interaction (PPI) network was conducted by STRING and Cytoscape, and the effect of HUB gene on the outcome of OC was analyzed.

**Results:**

A common pattern of up-regulation of miR-30a in OC was found. A total of 225 DEG, were identified, both OC-related and miR-30a-related. Many DEG are enriched in the interactions of intracellular matrix tissue, ion binding and biological process regulation. Among the 10 major Hub genes analyzed by PPI, five Hub genes were significantly related to the overall poor survival of OC patients, in which the low expression of ESR1,MAPK10, Tp53 and the high expression of YKT,NSF were related to poor prognosis of OC.

**Conclusion:**

Our results indicate that miR-30a is of significance for the biological progress of OC.

## Introduction

Common gynecological malignant tumors have the following categories: ovarian cancer (OC), cervical cancer, endometrial carcinoma, fallopian tube cancer, vulvar cancer and gestational trophoblastic tumor. Among them, although the incidence of OC is lower than that of cervical cancer and endometrial cancer, the lethal rate has far exceeded both, ranking first in gynecological malignant tumors. It is reported that more than 200,000 women worldwide suffer from OC, 125000 of whom die from it every year [1]. In the 2018 cancer statistics, 21,530 women in the United States were expected to be diagnosed with OC, and 13,980 died from OC [2]. It is difficult for patients to feel uncomfortable in OC’s early stage. And it is often at its advanced stage when patients feel it, accounting for 70% of all the cases of malignant ovarian tumors [3]. Chirlaque et al. [4] reported that between 2000 and 2004, the 5-year standardized net survival rate of ovarian cancer ranged from 36% in Spain to 42% in Belgium, with the net survival rate of young people much higher than that of the elderly. Between 1992 and 2004, the net survival rate increased in Belgium, France, Italy, Portugal, Spain and Switzerland, mainly of young and middle-aged women. However, the difference in 5-year net survival rates among these countries in 2004 was greater than that in 1992 [4]. It is obvious that the lethal rate of OC is high. If we can find a new target relating to the prognosis of OC, clarify its mechanism, and carry out targeted treatment for OC patients, it can not only significantly improve the clinical treatment effect and patients’ quality of life, but also play an irreplaceable role in monitoring cancer recurrence and guiding rehabilitation treatment.

MicroRNA (miRNA) is a kind of highly conserved non-coding small molecule RNA. At the post-transcriptional level of mRNA, it regulates gene expression through complete or incomplete complementary pairing with 3’UTR, CDS or promoter regions of mRNA to inhibit mRNA translation or directly degrade target mRNA [5]. As the miRNA family and its various regulatory functions were discovered, it is explicit that they are widely involved in physiological and pathological processes such as cell differentiation, proliferation and apoptosis, and are closely related to the occurrence and development of a variety of tumors [6, 7]. More and more studies have shown that the aberrant versions of hsa-miR-30a-5p (miR-30a) are involved in the biological process of a variety of tumors. Some studies have shown that the Lin28b/IRS1 axis targeting miR-30a-5p promotes the growth of colorectal cancer [8]. Liang et al. [9] believe that plasma miR-30a-5p can be used as a biomarker for early diagnosis and prognosis of lung cancer. Noori et al. [10] reported that microRNA-30a and its downstream Snail1 are related to the growth and metastasis of melanoma. The invasive growth associated with miR-30a is mainly mediated by the direct regulation of tumor metastasis and invasion related genes [11]. Besides, studies also found that miR-30a causes ovarian cancer through direct targeting and negative regulation of FOXD1 [12]. MiR-30a expression in ovarian cancer is significantly up-regulated [12, 13]. Therefore, based on the published data on miR-30a expression in OC,the objective of this study is to identify possible molecular targets and reveal the role of miR-30a by studying miRNAs expression, GEO and study review, and through bioinformatics analysis.

## Materials and methods

### Selection of GEO dataset

The gene microarray atlas of OC was obtained from the GEO database (http://www.ncbi.nlm.nih.gov/geo/). The following key words: (ovarian cancer OR ovarian carcinoma OR ovarian) and (microRNA OR non-coding RNA OR miRNA) are used for information retrieval. A microarray dataset of miR-30a expression in OC and normal ovarian tissue (NOT) was included.

### Study and data extraction of Mir-30a and ovarian Cancer in the literature

Through the literature retrieval on PubMed, Web of Science and Embase (till 1st, April, 2020), retrieval words are (microRNA-30 OR miRNA-30 OR miR-30 OR non-coding RNA OR microRNA-30a) AND (ovarian cancer OR ovarian carcinoma OR ovarian tumor). The inclusion and exclusion criteria are as follows: (1) study miR-30a expression in OC; (2) exclude reviews, non-clinical studies, case reports, meta-analysis and meeting summaries;(3) exclude those with on control groups.

### Gene ontology enrichment and target prediction analysis

The gene expression profiles of GSE18520,GSE14407 and GSE36668 come from GEO (http://www.ncbi.nlm.nih.gov/geo). The array data of GSE14407, GSE18520 and GSE36668 are respectively composed of 12, 53 and 4 OC samples; 12, 10 and 4 NOTS samples.

The Limma software package (version 3.6.3) in R/BioManager was conducted to distinguish the DEGs between OC and NOT. By default, the adjusted *P*-value (adj.*P*.Value) used Benjamini and Hochberg false discovery rate (FDR) to correct false-positive results. *P*<0.05 and |log2(FC)|>1 was set as the cut-off criterion. Based on the platform annotation file downloaded from the database, the probe data in the matrix file was converted into the gene symbols.

### Use Online Website

miRWALK3.0 (https://zmf.umm.uni-heidelberg.de/apps/zmf/mirwalk2/miRretsys-self.html) and TargetScan (https://www.targetscan.org/vert_72/) to predict the target gene of hsa-miR-30a-5p(TG_miRNA-30a-5p). Then, the gene overlap between the DEG integrated into OC was analyzed by bioinformatics software, and the TG_miRNA-30a-5p was predicted. The Bingo plug-in of Cytoscape software (version 3.7.2) was used to analyze the gene overlap by Gene Ontology (GO) and visualize it. The confidence score C ≥ 0.7 was set as the truncation standard. Then, the molecular complex (MCODE) was detected. PPI network module of cutoff = 2, node score cutoff = 0.2, k-core = 2 and max.depth = 100 were screened out.

### Survival analysis

Online tool Kaplan-Meier plotter (KM plotter, www.kmplot.com) can assess the impact of 21 cancers on survival rate. Those that have utmost impacts include the breast (nude 6234), ovarian (nasty 2190), lung (nasty 3452), and gastric (nasty 1440) cancer. OC patients were divided into the expression and low expression group, according to the median expression level of specific genes. Kaplan-Meier was used to analyze the overall survival of patients with OC. Calculate and show the hazard ratio (HR) of the 95% confidence interval (CI).

### Statistical analysis

The data were presented as mean ± standard deviation (SD). The difference was analyzed by two independent sample t-test between two groups. The correlation between miR-30a level and OC was analyzed by standardized mean difference (SMD) analysis with Stata 15.0 statistical software. Mantel-Haenszel formula (fixed-effects model) or DerSimonia-Laird formula (random-effects model) were used to combine and analyze different GEO data sets. When the Q statistic is significant (*p* ≤ 0.05 or I^2^ ≥ 50%), the random effect model is applied, otherwise the fixed effect model is applied. *P*<0.05 was considered that the difference was statistically significant.

## Results

### MiR-30a expression in OC based on GEO

**Fig. 1:**
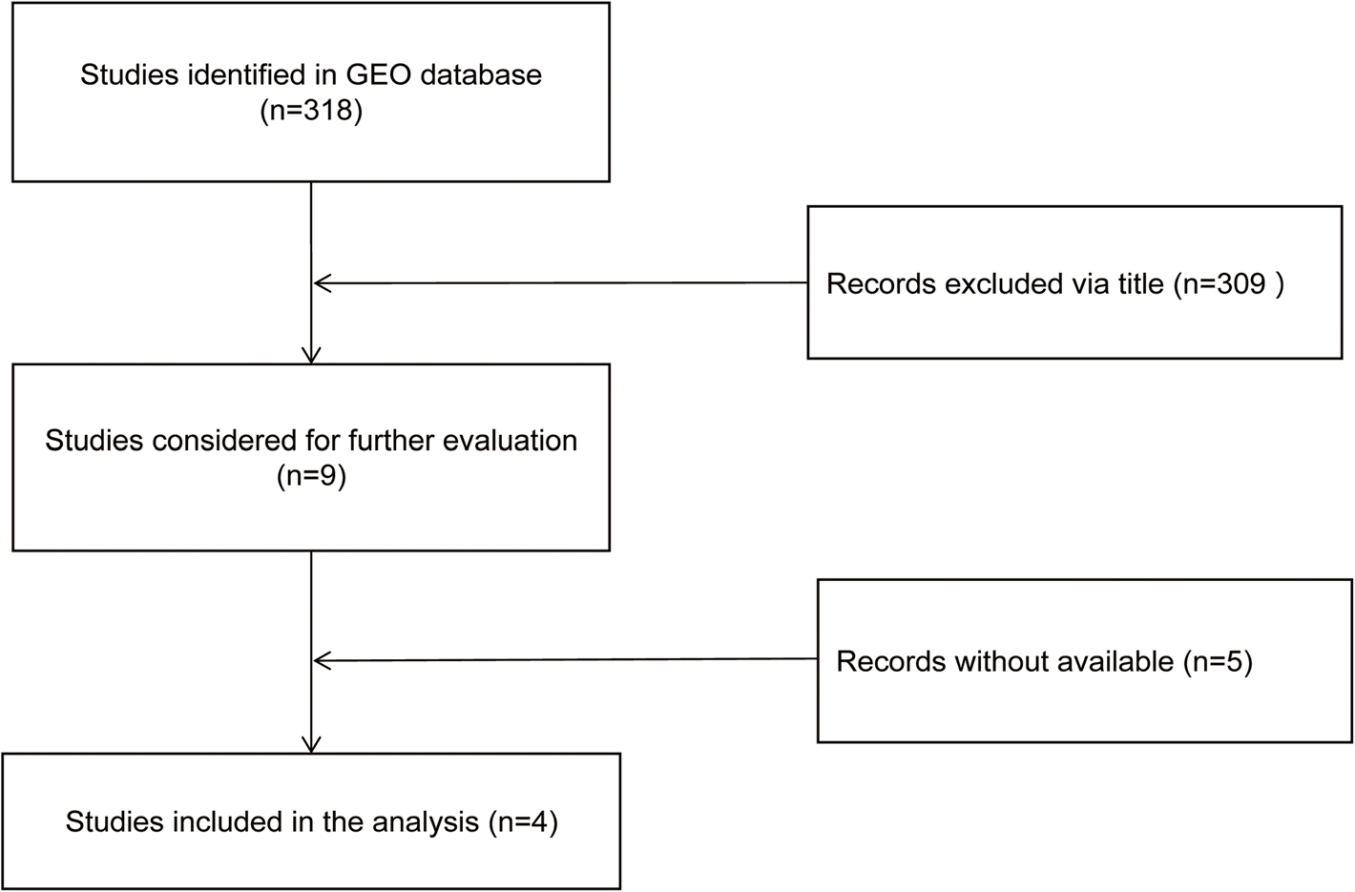
Research and screening flow Chart of GEO

**Fig. 2:**
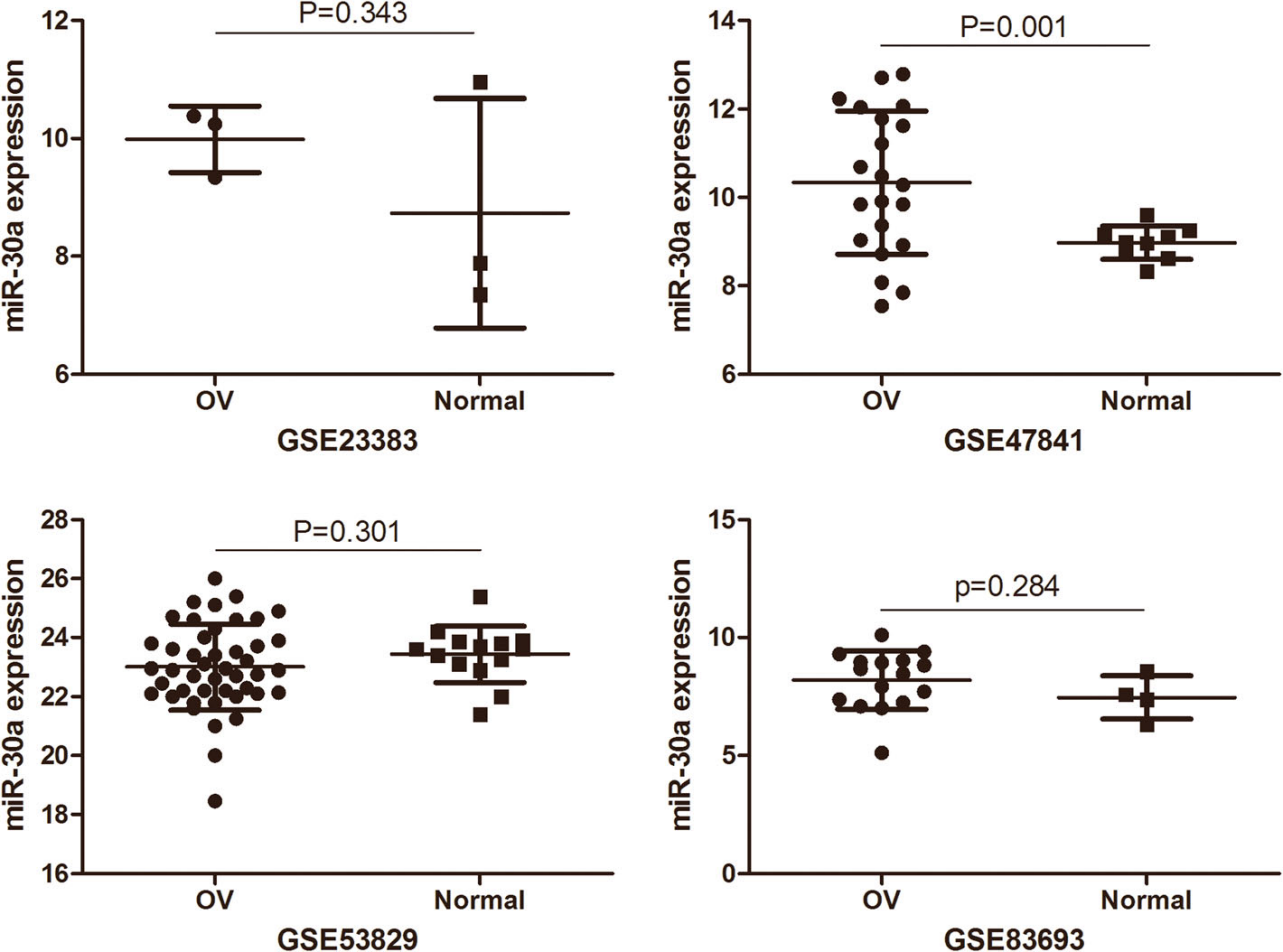
The expression of ovarian cancer miR-30a was concentrated in GEO data. OV: ovarian cancer tissue; Normal: normal ovarian tissue; miR-30a: hsa-miR-30-5p

**Fig. 3:**
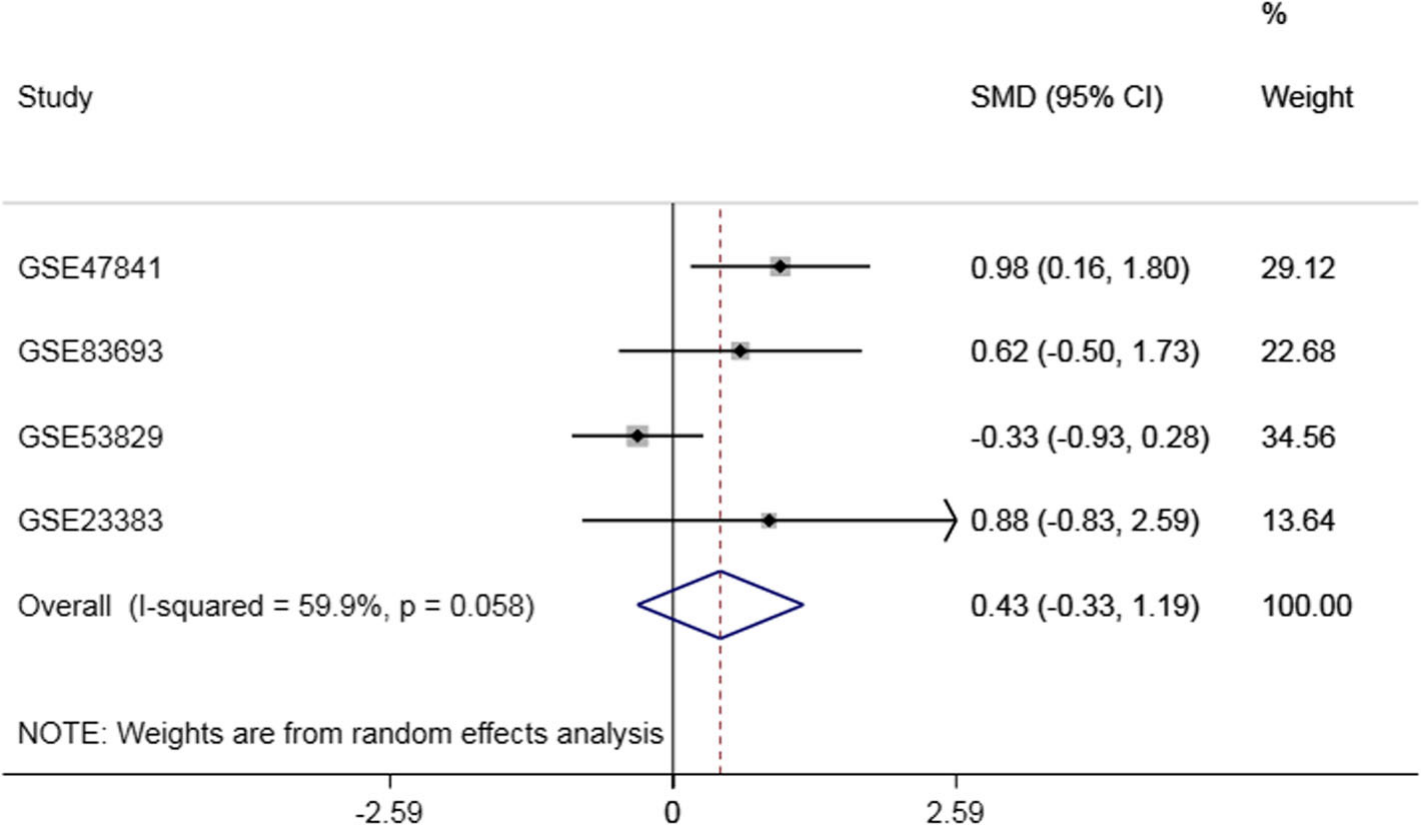
Forest plot of differential expression of miR-30a Gene in GEO data set

Based on the GEO dataset (Fig. 1), the expression of miR-30a is accessed in a series of OC and NOT. In this study, four GEO data sets (GSE83693, GSE47841, GSE53829 and GSE23338) were gathered. In the GSE47841 dataset, the miR-30a expression of miR-30 in OC tissue was higher than that in the NOTS (P<0.01) Compared with NOTs group. While the miR-30 expression of miR-30a in OC tissue increased in the GSE23338 and GSE83693 dataset, and the miR-30a expression of miR-30a in OC tissue decreased in GSE53829 dataset, but all *P*-values were greater than 0.05. The research features were listed in Table 1 and Fig. 2.However, there was no significant difference between OC and NOT (SMD = 0.43,95%CI:-0.33 ~ 1.19,*P* = 0.264). The results of the forest plots were shown in Fig. 3.

**Table 1:** Basic characteristics of miR-30a related Research on Ovarian Cancer in GEO data set

Study	Ovarian cancer tissue			Normal ovarian tissue			t	*p*
	Mean	SD	n	Mean	SD	n		
GSE47841	10.33	1.62	21	8.97	0.38	9	3.622	0.001
GSE83693	8.2	1.23	16	7.47	0.92	4	−1.11	0.284
GSE53829	23	1.45	45	23.44	0.95	14	1.05	0.301
GSE23383	9.99	0.57	3	8.73	1.94	3	1.08	0.343
Total	SMD(95%CI) = 0.43(−0.33 ~ 1.19),*p* = 0.264,I^2^ = 59.9%,*p* = 0.058

### Expression profile of miR-30a in OC and NOTS

**Fig. 4:**
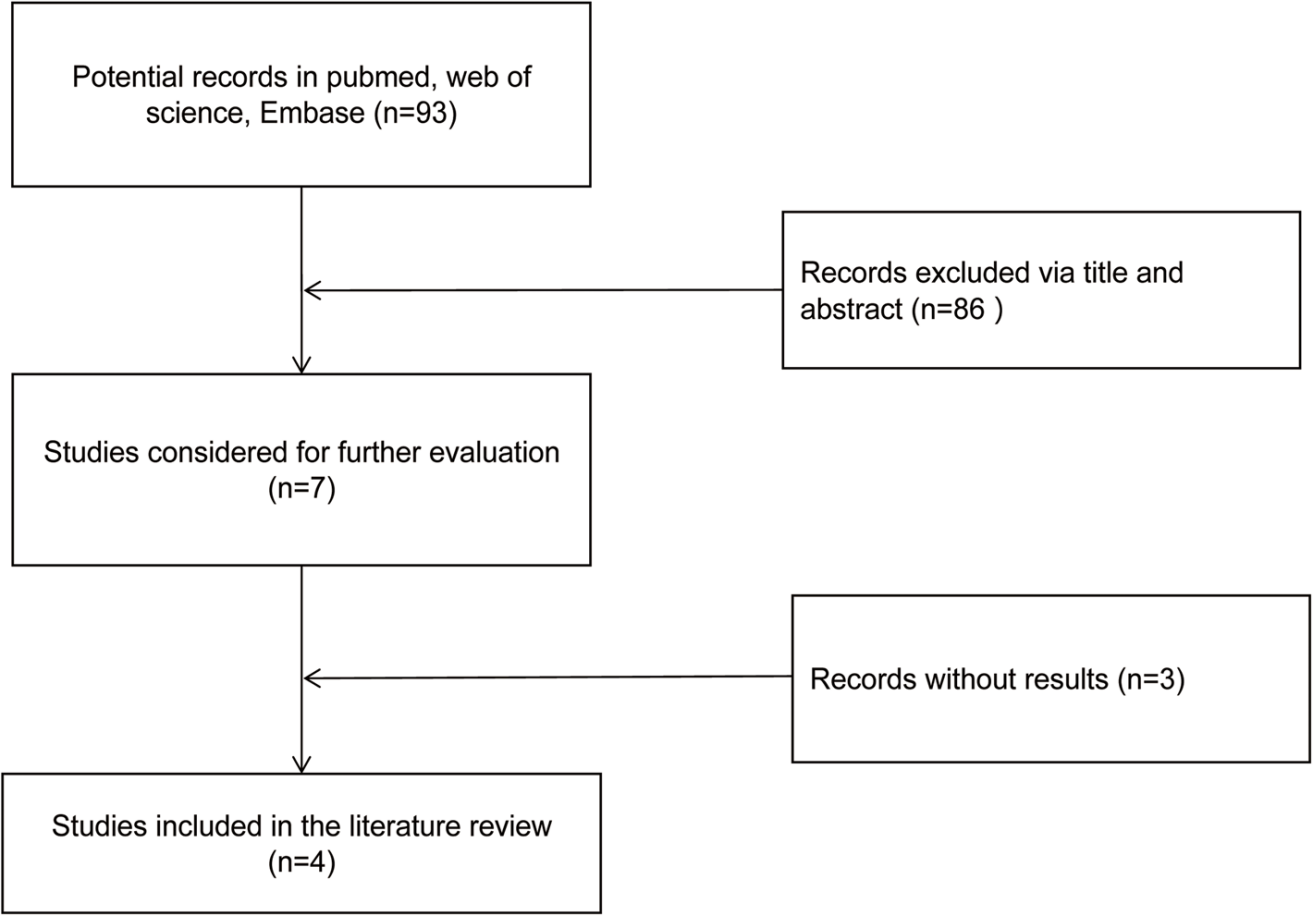
Screening of literatures on the expression of miR-30a in ovarian cancer

Then, the expression of miR-30a in ovarian cancer was studied according to the literature data. As shown in Fig. 4, four studies [12–15] up to selection criteria were selected from the literature. Three out of the four studies showed that the expression level of miR-30a in OC tissues was significantly higher than that in non-OC tissues, while in the study, compared with well differentiated ovarian cancer, the level of miR-30a in poorly differentiated ovarian cancer group was significantly higher (Table 2).

**Table 2:** Basic characteristics of search literatures related to miR-30a in ovarian cancer

Author	Year	Country	Case		Control		Result	Detection methods
			Source name	n	Source name	n		
Wang Y	2013	China	low-grade OPSC	16	high-grade OPSC	53	up-regulation	qRT-PCR
Zhou J	2015	China	Ovarian cancer urine	39	Urine of HP	30	up-regulation	qRT-PCR
Wang Y	2018	China	HGSOC	11	NFT	10	up-regulation	qRT-PCR
Závesky	2018	Czech Republic	OCPF	26	Blood of HP	34	up-regulation	qRT-PCR

Note:OPSC: ovarian papillary serous carcinoma;HSSOC:High-grade serous ovarian carcinoma;NFT:normal fallopian tube;OCPF:Ovarian cancer peritoneal fluid;HP:healthy people

### miR-30a prediction and bioinformatics analysis; data preprocessing and DEGS screening

**Fig. 5:**
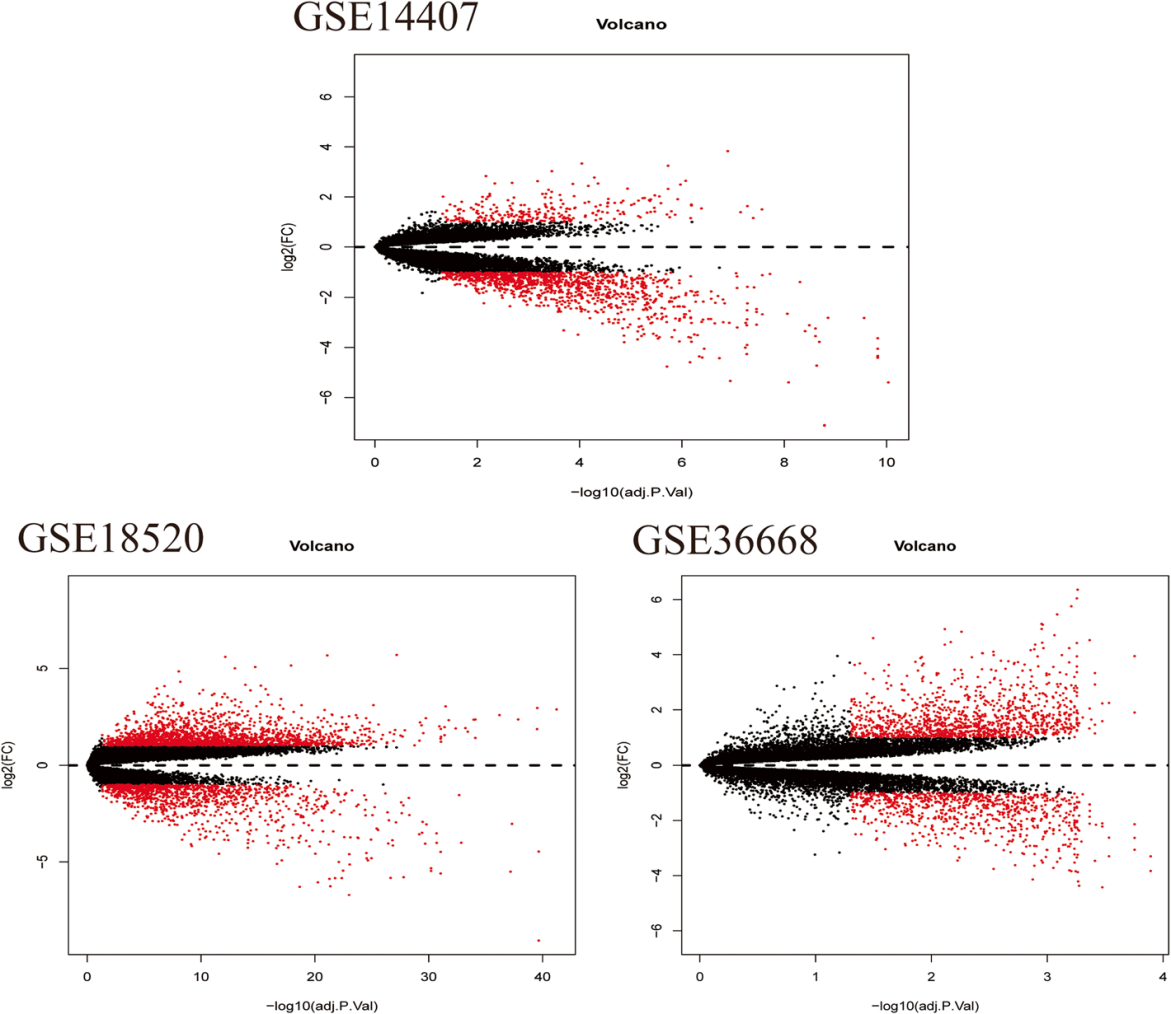
Volcano Map of Genome-wide mRNA Map detected by three Ovarian Cancer-related data sets from GEO. Aberrantly expressed mRNAs with *P* < 0.05 and |log(FC)| > 1 were represented by red. up-regulated genes were indicated by red plots above, down-regulated genes were indicated by red plots below,and normally expressed mRNAs were indicated by black plots . The x-axis represents the adj.P of-log10. The associated strength is expressed by the *P* value of each mRNA.adj.P.Value, adjusted *P* value; FC, fold change

**Fig. 6:**
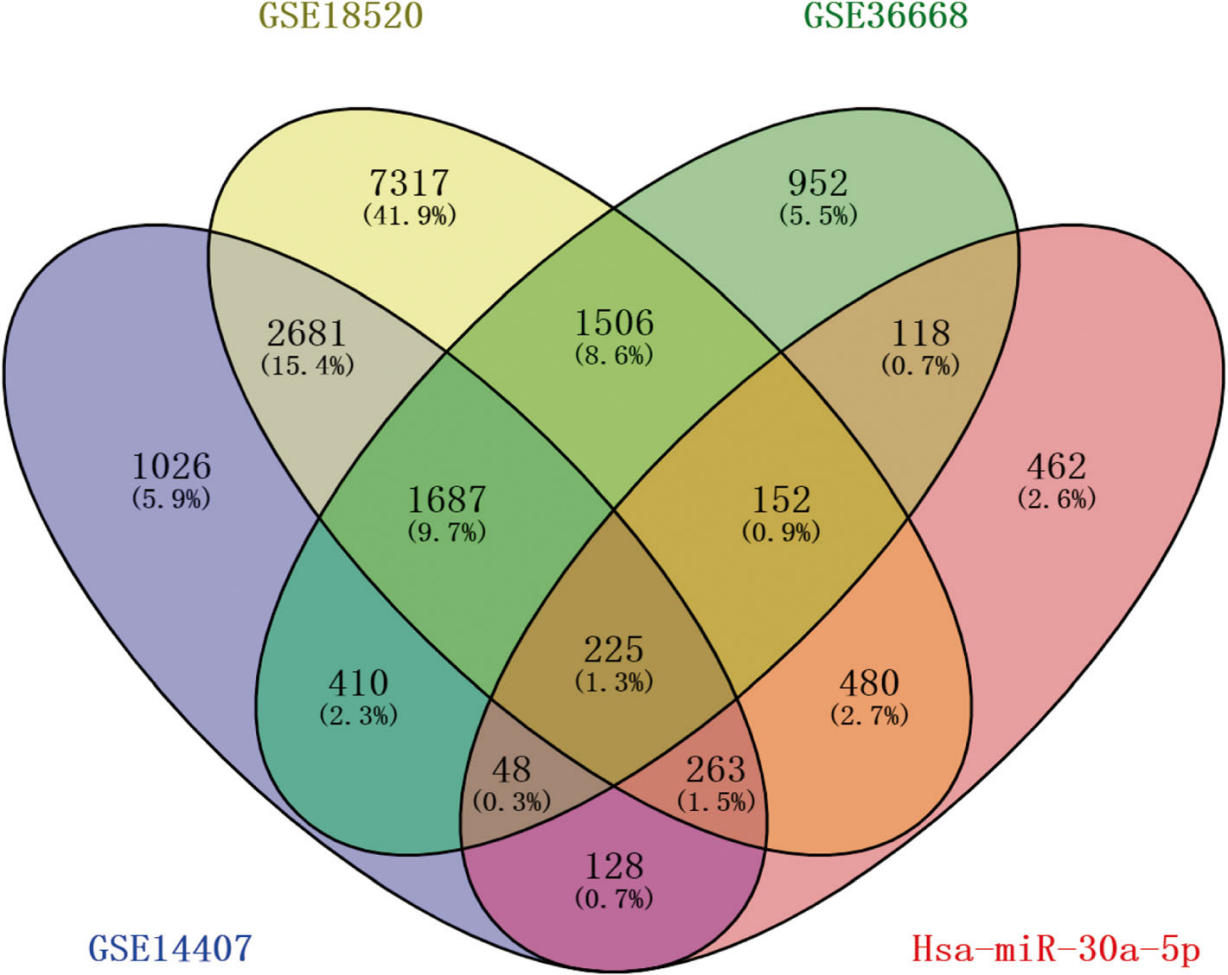
The Venn map of hsa- mir-30-5p related differentially expressed genes in TG_miR-182-5p and other three GEO data sets, and the overlapping region represents the recognized DEGs. DEGs: differentially expressed genes; TG_miR-30a-5p, target genes of hsa-miRNA-30-5p

6468, 14,311 and 5098 DEG were identified from GSE14407, GSE18520 and GSE36668 datasets respectively. The histological types of ovarian cancer in GSE14407, GSE18520 and GSE36668 data sets were ovarian epithelial cell carcinoma, ovarian epithelial cell carcinoma and ovarian serous carcinoma, respectively.1912 common DEG were filtered out in these three datasets (Figs. 5 and 6) by Venny 2.1.0 (https://bioinfogp.cnb.csic.es/tools/venny/). Then, 1312 TG_hsa-miR-30a-5p, were predicted based on miRWALK3.0 and TargetScan, of which 225 were verified in 1876 common DEG. According to the gene expression data overview, there are 111 up-regulated hsa-miR-30a-5p-related genes and 114down-regulated hsa-miR-30a-5p-related genes in OC tissues compared with non-OC tissues (Fig. 6, Table 3).

**Table 3:** differentially expressed genes of hsa- mir-30a-5p in ovarian cancer tissues and normal ovarian tissues in GSE18520 dataset (Top 10)

DEG	logFC	*P*.Value	adj.P.Val
up-regulated genes			
GLDC	3.879464906	9.08E-12	6.62E-11
MAL	4.080880545	4.09E-08	1.59E-07
PRSS21	2.700693431	1.47E-11	1.04E-10
GRHL2	2.41672613	1.49E-27	3.11E-25
CTHRC1	2.333368517	1.48E-04	3.27E-04
EYA2	2.253746336	1.92E-05	4.86E-05
SOX9	2.077258364	0.000000019	7.85E-08
E2F7	1.961647476	5.66E-15	7.90E-14
KIAA0101	1.916963173	4.12E-15	5.93E-14
SBK1	1.887544551	6.62E-12	4.95E-11
down-regulated genes			
GFPT2	−3.731162519	4.91E-26	7.51E-24
ADAMTS3	−3.417352741	4.99E-12	3.83E-11
WNT5A	−3.0965058	1.44E-08	6.06E-08
HLF	−2.953607331	1.97E-30	7.46E-28
DPYSL2	−2.904638189	1.50E-07	5.35E-07
NR3C2	−2.607594668	7.07E-13	6.35E-12
ADRA2A	−2.557257353	1.78E-13	1.83E-12
TCF21	−2.488576443	2.04E-22	1.27E-20
ABI3BP	−2.261098823	4.93E-20	1.92E-18
POLI	−2.238370149	2.41E-16	4.43E-15

### Functional analysis of miR-30a-related DEGs in OC

**Fig. 7:**
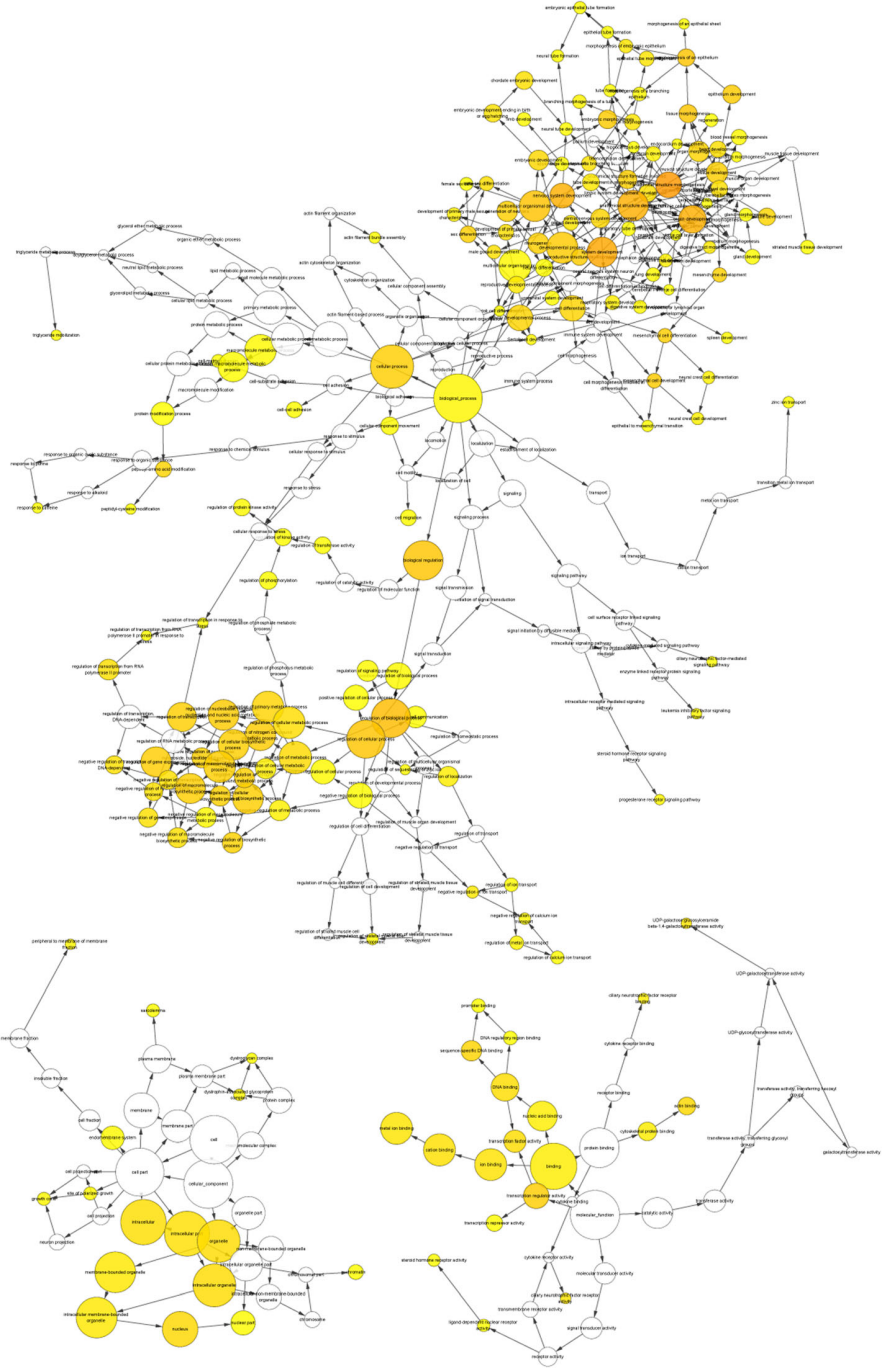
Results of GO enrichment Analysis of miR-30a Ovarian Cancer Target Gene. The yellow circle represents functional enrichment, and the larger the circle, the darker the color, the more genes are enriched in this pathway. The connecting lines represent the association between gene and gene

The functional enrichment analysis is carried out by Bingo plug-in in Cytoscape. The three most important enrichments of molecular function, biological pathway and cell composition were shown in Table 4. The analysis showed that many target genes participated in biological processes such as biological regulation, ion binding and intracellular matrix tissue (Fig. 7). We found that SOX9, transcription factor 21 (TCF21) and Wnt-5a and other genes play a significant role in these key enrichment pathways.

**Table 4:** Functional enrichment Analysis of hsa-mir-30a-5p related differentially expressed genes in Ovarian Cancer

Term	Description	Count	FDR
Biological processes			
GO:0009987	cellular process	192	0.003
GO:0065007	biological regulation	169	0.00017
GO:0050789	regulation of biological process	163	0.00016
Molecular Function (Go)			
GO:0005488	binding	175	2.91E-05
GO:0043167	ion binding	99	0.0018
GO:0097159	organic cyclic compound binding	91	0.0017
Cellular Component			
GO:0005622	intracellular	190	0.0033
GO:0044424	intracellular part	189	0.0031
GO:0043226	organelle	174	0.0031

### PPI network construction and modules selection

**Fig. 8:**
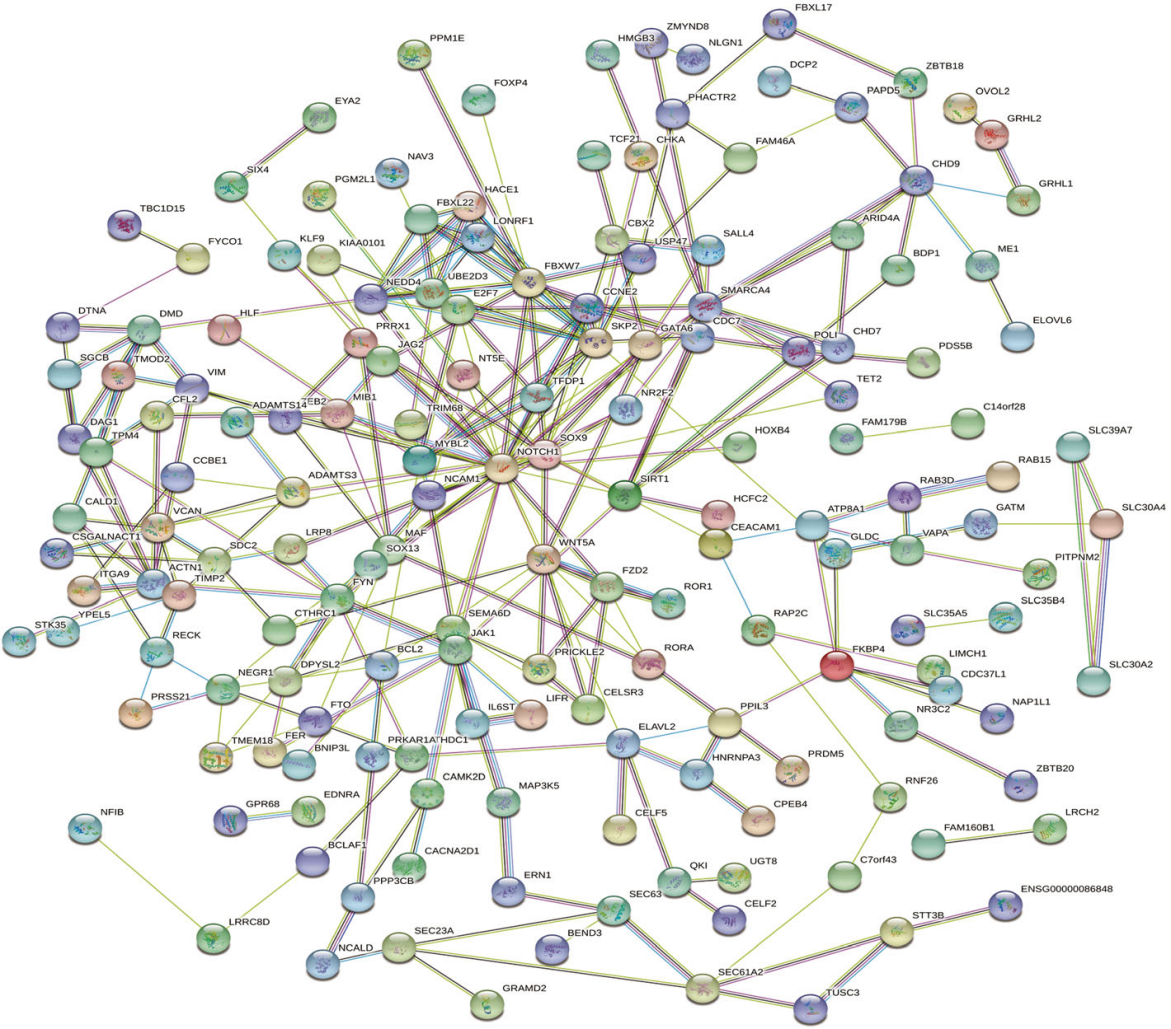
Protein-protein interaction network of hsa-miR-30a-5p-related DEGs. The lines represent interaction relationship between nodes. DEGs, differentially expressed genes. The line between the circle nodes represents the interaction between the two proteins linked by the line. Colored nodes:query proteins and first shell of interactors;white nodes:second shell of interactors;empty nodes:proteins of unknown 3D structure;filled nodes:some 3D structure is known or predicted.

**Fig. 9:**
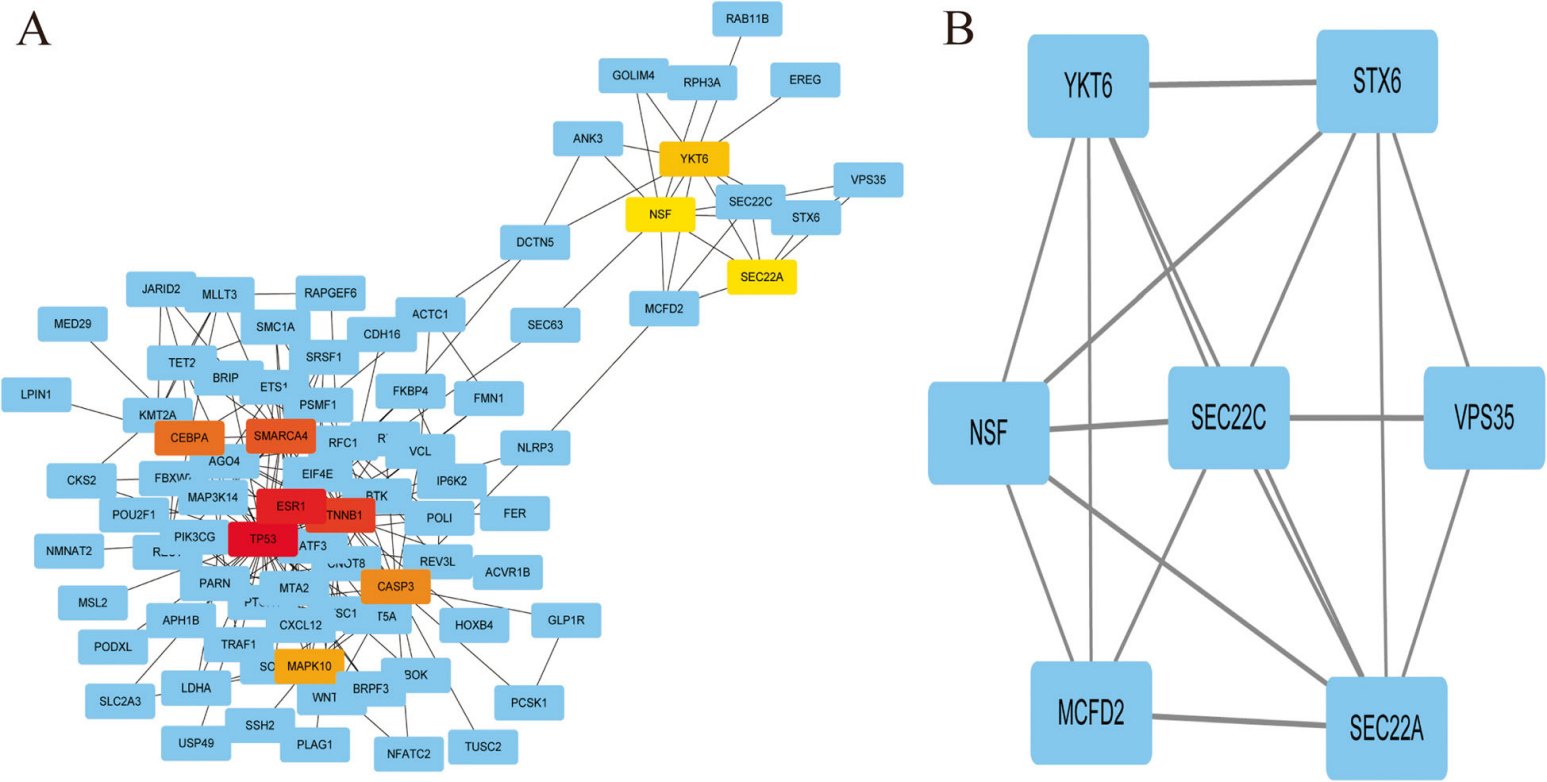
Protein-protein interaction network. **a**: Protein-protein interaction network of hube genes of hsa-miR-30a-5p-related DEGs. **b**: A significant module selected from protein-protein interaction network of hsa-miR-30a-5p-related DEGs. Red and yellow represent the key Hub genes of Tp10. The darker the color, the stronger the association with other genes in the PPI network. The lines represent interaction relationship between nodes. DEGs,differentially expressed genes

The PPI network of miR-30a-related DEG consists of 225 nodes and 287 edges, including 111 up-regulated genes and 114 down-regulated genes (Fig. 8). Setting ≥10 degrees as the truncation criteria, 10 genes were selected as Hub genes, and there was a close correlation between HUB genes (Fig. 9a). By using MCODE, an important module was obtained from the PPI network of miR-30a-related DEG, including 7 nodes and 17 edges (Fig. 9b).

### Survival analysis

**Fig. 10:**
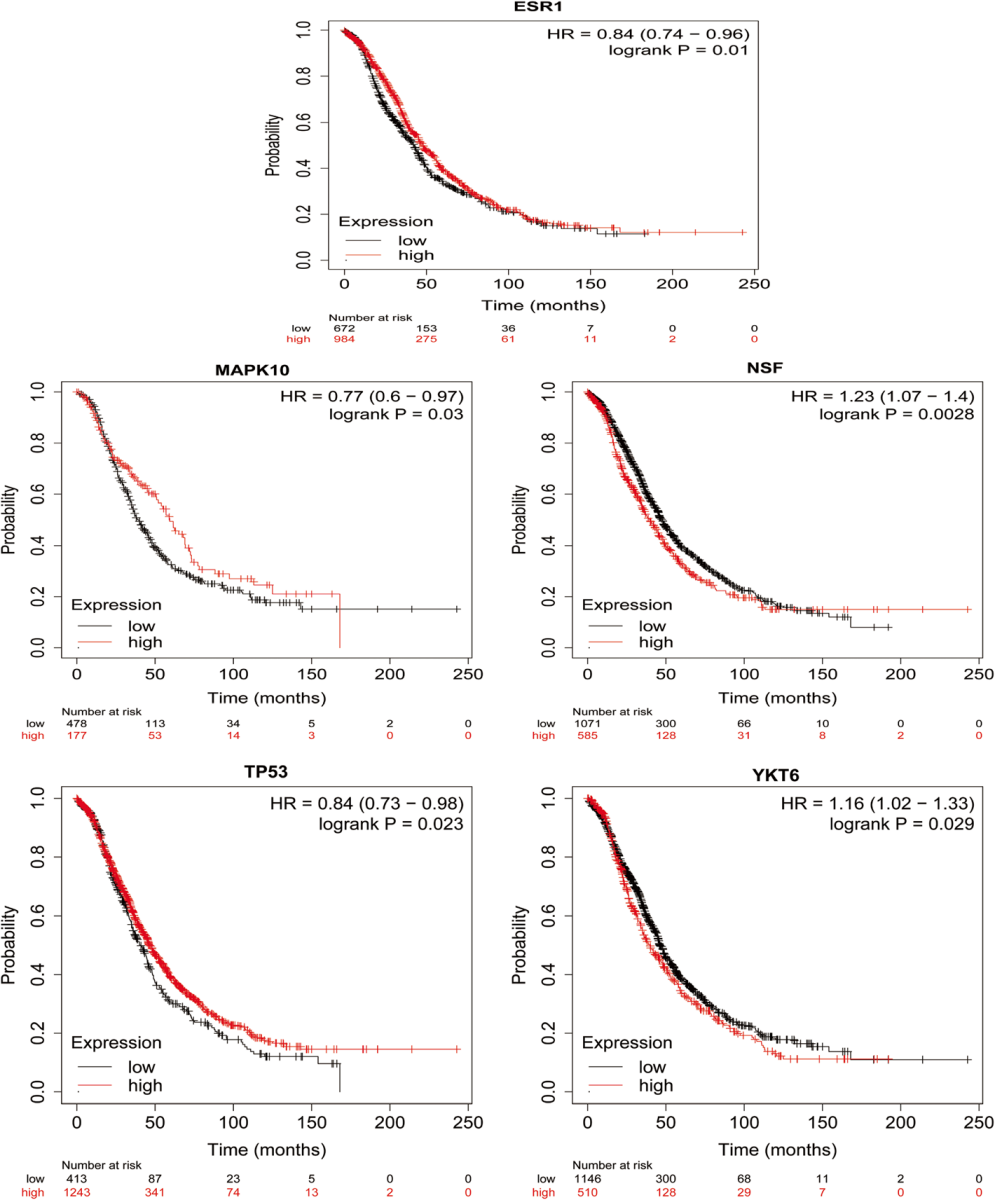
The expression of ESR1, MAPK10, Tp53, YKT, NSF and the prognosis of patients with ovarian cancer were analyzed. According to the median expression level, patients with ovarian cancer were divided into high expression group and low expression group

The prognostic value of 10 HUB genes in the PPI network was accessed on the www.kmplot.com website. According to the high and low expression of each HUB gene,we analyzed the overall survival of patients with OC. The results showed that low expression of ESR1 (HR = 0.84,95%CI:0.74 ~ 0.96,*P* = 0.01), low expression of MAPK10 (HR = 0.77,95%CI:0.60 ~ 0.97,*P* = 0.03), low expression of Tp53 (HR = 0.84,95%CI:0.73 ~ 0.98,*P* = 0.023), high expression of YKT6 (high expression of HR = 1.16,95%CI:1.02 ~ 1.33,*P* = 0.029), and high expression of NSF (HR = 1.23,95%CI:1.07 ~ 1.40,*P* = 0.003) were associated with overall survival decreasing of patients with ovarian cancer (Fig. 10).

## Discussion

In this study, based on the data of GEO dataset and published studies, we identified the aberrant expression of miRNA related to OC by comparing the expression profiles of miRNA in OC and NOTS tissues. In addition, through GO analysis, PPI network and Kaplan-Meier plotter, the new markers and potential targets of miR-30a took part in the regulation of key biological processes of OC were identified and analyzed.

By far, few researches have been done on the characteristics of miR-30a in OC. In 2014, the result of Zhou et al. [13] showed the level of miR-30a expression in urine in OC was higher than that of healthy controls.

Some studies showed that miR-30a expression was up-regulated in the tissues of ovarian cancer [12, 15]. Interestingly, the study of, Wang et al. [14] showed that the level of miR-30a expression in poorly differentiated ovarian papillary serous carcinoma tissues was obviously higher than that in well-differentiated ovarian papillary serous carcinoma tissues. According to the GEO dataset, only the GSE47841 dataset showed an increase in miR-30a expression in OC, while other datasets showed no statistical difference. As the expression level of miR-30a in OC is still controversial, further studies are needed to clarify the role of miR-30a in OC.

MiR-30a is one of the quite important miRNAs in OC regulation. Lee et al. [16] suggested that miR-30a expression was noteworthily increased in OC, and the high level of miR-30a expression was related to the significant shortening of disease-free survival. In addition, Gong et al. [16] found that TMED2 is an oncogene and a potential target for the treatment of epithelial ovarian cancer, while TMED2 increases the expression of IGF1R by competing with miR-30a. The study of Han et al. [17] showed that the feedback loop between miR-30a-5p and DNMT1 mediated the resistance of ovarian cancer cells to cisplatin. Liu et al. [18] found that high expression of miRNA-30a-5p can promote cell growth and colony formation, and enhance cell migration and invasion. Therefore, miRNA-30a-5p is expected to become a significant new target for drug resistance treatment of ovarian cancer. Given the current situation, it is necessary to further clarify the molecular mechanism and clinical importance relating to the aberrant expression of miR-30a in ovarian cancer.

ESR1 encodes the estrogen receptor, a ligand-activated transcription factor, which is composed of the binding of several important hormones, DNA binding and the important domain of transcriptional activation. Estrogen and its receptors play an important role in hypoplasia and reproductive function, and are also involved in pathological processes including breast cancer, endometrial cancer and osteoporosis [19]. MAPK10, also known as JNK3, belongs to the subgroup of JUN N-terminal kinase (JNK) in the mitogen-activated protein kinase (MAPK) family. The study of Silvia et al. [20] showed that the prognosis of ovarian cancer patients with low expression of ESR1 was poor compared with that of high expression. This is consistent with the conclusion of this study. Dai et al. [21] reported that the expression of MAPK10 protein was negatively correlated with the overall survival time of OC. It is not consistent with the conclusion of this study. In this study, the survival analysis of Hub gene relating to miR-30a target gene showed that the expression of MAPK10 protein was low and the overall survival time of OC was significantly shortened. MAPK10 is inclined to be considered as a tumor suppressor gene in current studies [22], and more studies are needed to verify it. The Tp53 gene encodes a tumor suppressor protein that includes transcriptional activation, DNA binding and oligomerization domains. The coding protein regulates the expression of target genes in response to a variety of cellular stress, thereby inducing cell cycle arrest, apoptosis, senescence, DNA repair or metabolic changes [23]. Hurley et al. [24] reported that the sensitivity and specificity of the TP53 and PAX8 joint detection of ovarian cancer were 56 and 98%, respectively. The study of Chen et al. [25] showed that ubiquitin ligase TRIM71 inhibits the occurrence of ovarian tumors by degrading mutant In this study, the survival analysis of Hub gene relating to miR-30a target gene showed that the low expression of Tp protein was closely related to the poor prognosis of OC. YKT6, also known as synaptic vesicle protein homologue YKT6 precursor, is a protein encoded by YKT6 gene. Marc et al. [26] reported that patients with non-small cell lung cancer with high expression of YKT6 protein had shorter both disease-free survival and overall survival. In this study, the survival analysis of Hub gene relating to miR-30a target gene showed when YKT6 protein was highly expressed, the prognosis of OC patients was poor.

N-ethylmaleimide-sensitive factor (NSF) is an oligomeric protein with a molecular weight of 76KDa, an ATP enzyme involved in membrane fusion [27]. Lee et al. [28] studies have shown that the overall survival rate of colorectal cancer patients with nuclear dominant expression of GS28 (Golgi snare protein,28 kDa), a member of the NSF attachment protein receptor family, is significantly lower than that of patients with non-nuclear dominant expression. The results of this study showed when the expression of NSF was high, the prognosis of patients with OC was poor. Thus, YKT6, NSF is closely related to OC and is expected to become a new prognostic marker of OC.

In the current study, we found that miR-30a-mediated DEGs are involved in the regulation of key biological processes of ovarian cancer, such as SOX9, transcription factor 21 (TCF21), Wnt-5a,etc. Transcription factor Sox9 belongs to the HMG protein family and is related to early ovarian development [29]. Previous studies have shown that SOX9 plays a needful role in tumorigenesis and metastasis. Siu et al. [30] reported that the migration and invasion of ovarian cancer cells were regulated by hexokinase 2 through FAK/ERK1/2/MMP9/NANOG/SOX9 signal pathway. Raspaglio et al. [31] found that SOX9 expression is up-regulated in OC and is related to the poor prognosis of the controls. Our current study shows that the level of SOX9 mRNA expression in OC is higher than that in NOTS. We assume that SOX9 is a cancer-promoting factor. TCF21 is a recently discovered new tumor suppressor factor, which is widely expressed in interstitial cells or their derived tissue cells during the development of cardiovascular system, genitourinary system and respiratory system, and has a great influence on cell growth and differentiation [32]. Duan et al. and others [33] found that TCF21 can target PI3K/Akt and ERK signal pathways, thus inhibiting tumor-associated angiogenesis and cholangiocarcinoma growth. Many pieces of evidence show that TCF21 is regulated by miRNAs, such as miR526b [34], miR-205 [35], which can inhibit tumor development. Zhou et al. [36] found that microRNA-30-3p targeting TCF21 inhibited endothelial cell injury induced by inflammatory factors. Based on the above, we speculate that TCF21 may be the target gene of miR-30a in OC. Wnt ligands belong to a family of at least 19 secretory proteins, which play a critical role in cell differentiation, proliferation and histogenesis. Wnt-5a is one of the quite important factors in the non-classical pathway of Wnt signaling, which can activate messy proteins through tuberculosis of receptors on the cell membrane, and thus promote proliferation like cell polarity establishment and cytoskeleton rearrangement [37]. Arabzadeh et al. [38] reported that in human ovarian cancer cell line SKOV-3, compared with normal controls, the expression of WNT5A was significantly decreased and had immunomodulatory activity. It is consistent with our current research.

In conclusion, Our study indicated that miR-30a is of importance in OC biology. However, further in vivo and in vitro researches are needed to study its pathogenesis in order to verify the key role of molecular networks regulated by miR-30a in OC.

## Data Availability

The datasets used and/or analysed during the current study are available from the corresponding author on reasonable request.

## Abbreviations

MiRNAs: MicroRNAs
miR-30a: Has-miR-30a-5p
GEO: Gene Expression Omnibus
OC: Ovarian cancer
DEG: Differentially expressed gene
GO: Gene Ontology
FDR: False discovery rate
SMD: Standardized mean difference
HR: Hazard ratio
NOT: Normal ovarian tissue
JNK: JUN N-terminal kinase
MAPK: Mitogen-activated protein kinase
NSF: N-ethylmaleimide-sensitive factor
TCF21: Transcription factor 21
